# Use of longer sized screws is a salvage method for broken pedicles in osteoporotic vertebrae

**DOI:** 10.1038/s41598-020-67489-2

**Published:** 2020-06-26

**Authors:** Ming-Kai Hsieh, Mu-Yi Liu, Jin-Kai Chen, Tsung-Ting Tsai, Po-Liang Lai, Chi-Chien Niu, Ching-Lung Tai

**Affiliations:** 1 Institute of Biotechnology, National Taiwan University, Taipei, Taiwan; 2 Bone and Joint Research Center, Chang Gung Memorial Hospital, Taoyuan, Taiwan; 3 Department of Orthopaedic Surgery, Spine Section, Chang Gung Memorial Hospital and College of Medicine, Chang Gung University, Taoyuan, Taiwan; 4 Ph.D. Program in Biomedical Engineering, College of Engineering, Chang Gung University, Taoyuan, 33302 Taiwan; 5 Graduate Institute of Biomedical Engineering, Chang Gung University, Taoyuan, Taiwan

**Keywords:** Medical research, Engineering

## Abstract

Screw loosening due to broken pedicles is a common complication resulting from the insertion of screws either with inadequate diameters or into an osteoporotic pedicle. In this novel in vitro study, we tried to clarify the contribution of the pedicle to screw fixation and subsequent salvage strategies using longer or larger-diameter screws in broken pedicles. Sixty L4 fresh-frozen lumbar vertebrae harvested from mature pigs were designed as the normal-density group (n = 30) and decalcified as the osteoporosis group (n = 30). Three modalities were randomly assigned as intact pedicle (n = 30), semi-pedicle (n = 15), and non-pedicle (n = 15) in each group. Three sizes of polyaxial screws (diameter × length of 6.0 mm × 45 mm, 6.0 mm × 50 mm, and 6.5 mm × 45 mm) over five trials were used in each modality. The associations between bone density, pedicle modality and screw pullout strength were analyzed. After decalcification for 4 weeks, the area bone mineral density decreased to approximately 56% (*p* < 0.05) of the normal-density group, which was assigned as the osteoporosis group. An appropriate screw trajectory and insertional depth were confirmed using X-ray imaging prior to pullout testing in both groups. The pullout forces of larger-diameter screws (6.5 mm × 45 mm) and longer screws (6.0 mm × 50 mm) were significantly higher (*p* < 0.05) in the semi- and non-pedicle modalities in the normal-density group, whereas only longer screws (6.0 mm × 50 mm) had a significantly higher (*p* < 0.05) pullout force in the non-pedicle modalities in the osteoporosis group. The pedicle plays an important role in both the normal bone density group and the osteoporosis group, as revealed by analyzing the pullout force percentage contributed by the pedicle. Use of a longer screw would be a way to salvage a broken pedicle of osteoporotic vertebra.

## Introduction

Pedicle screw fixation during spinal surgery has been a common procedure for several decades^1–3^. Screw loosening due to broken pedicles is a common complication resulting from the insertion of screws either with inadequate diameters or into an osteoporotic pedicle^4–7^. In a well-designed L4 sawbone model^4^, the pedicle contributes to the screw pullout strength, and only 29–39% of the pullout strength is retained after the pedicle breaks. However, there is a lack of biomechanical studies addressing the pullout strength of broken pedicles under normal bone density and osteoporosis conditions that are more clinically practical. Biomechanical testing has shown a decrease of 80.7% in elastic modulus, 74.7% in yield stress and 74.5% in ultimate stress in decalcified vertebral bodies compared with non-decalcified bodies^8^, but the strength of the pedicle cannot be evaluated only by the vertebral body strength. Varghese et al. used polyurethane foam blocks to investigate the effects of bone density, insertion depth and insertion angle on pedicle screw pull out strength and insertion torque. The contribution of bone density was found to be the most influential factor for the pullout strength and insertion torque^9,10^; however, their result was hardly convincing, as polyurethane foam blocks were used instead of an anatomical vertebral model.

To the best of the authors’ knowledge, no existing research has addressed the effect of the absence of pedicles on screw anchoring power using different-density porcine vertebrae rather than test blocks or simulated sawbones. The present study is the first to determine any difference in pullout strength on the fixation stability of pedicle screws in different broken pedicle modalities and under different bone densities.

In this novel in vitro experimental study, we compared the pullout strength of screws between the intact pedicle, semi-pedicle and non-pedicle modalities in normal and osteoporotic porcine vertebral models and then examined subsequent salvage strategies.

We hypothesized that the pedicle contributes to the screw pullout strength in both the normal bone density group and the osteoporosis group, and both longer and larger-diameter screws could be used for salvage methods.

## Results

### BMD during specimen decalcification

**Figure 1:**
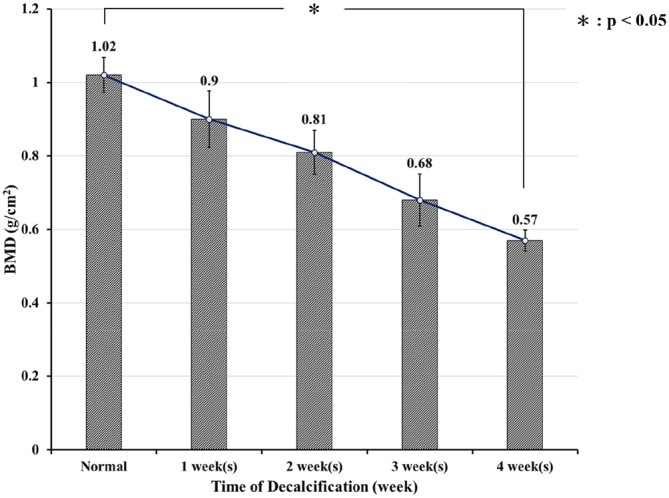
The serial BMD changes from normal to weekly decalcification. The values were 1.02 ± 0.04 g/cm^2^ in the normal group, 0.9 ± 0.07 g/cm^2^ in the first week, 0.81 ± 0.05 g/cm^2^ in the second week, 0.68 ± 0.07 g/cm^2^ in the third week and 0.57 ± 0.02 g/cm^2^ in the fourth week. Only 56% (*p* < 0.01) of the normal vertebral BMD was retained after 4 weeks of decalcification.

The serial changes in area BMD from normal to decalcified states are shown in Fig. 1. The serial area BMD was recorded as 1.02 ± 0.04 g/cm^2^ in the normal group, 0.9 ± 0.07 g/cm^2^ in the first week, 0.81 ± 0.05 g/cm^2^ in the second week, 0.68 ± 0.07 g/cm^2^ in the third week and 0.57 ± 0.02 g/cm^2^ in the fourth week. After treatment with EDTA for 4 weeks, the area BMD decreased to approximately 56% (p < 0.05) of that of the normal vertebrae and reached the osteoporotic stage^8^. Specimens that had been treated with EDTA for 4 weeks were assigned to the osteoporosis group, and those without EDTA treatment were assigned to the normal-density group.

### Image analysis

**Figure 2:**
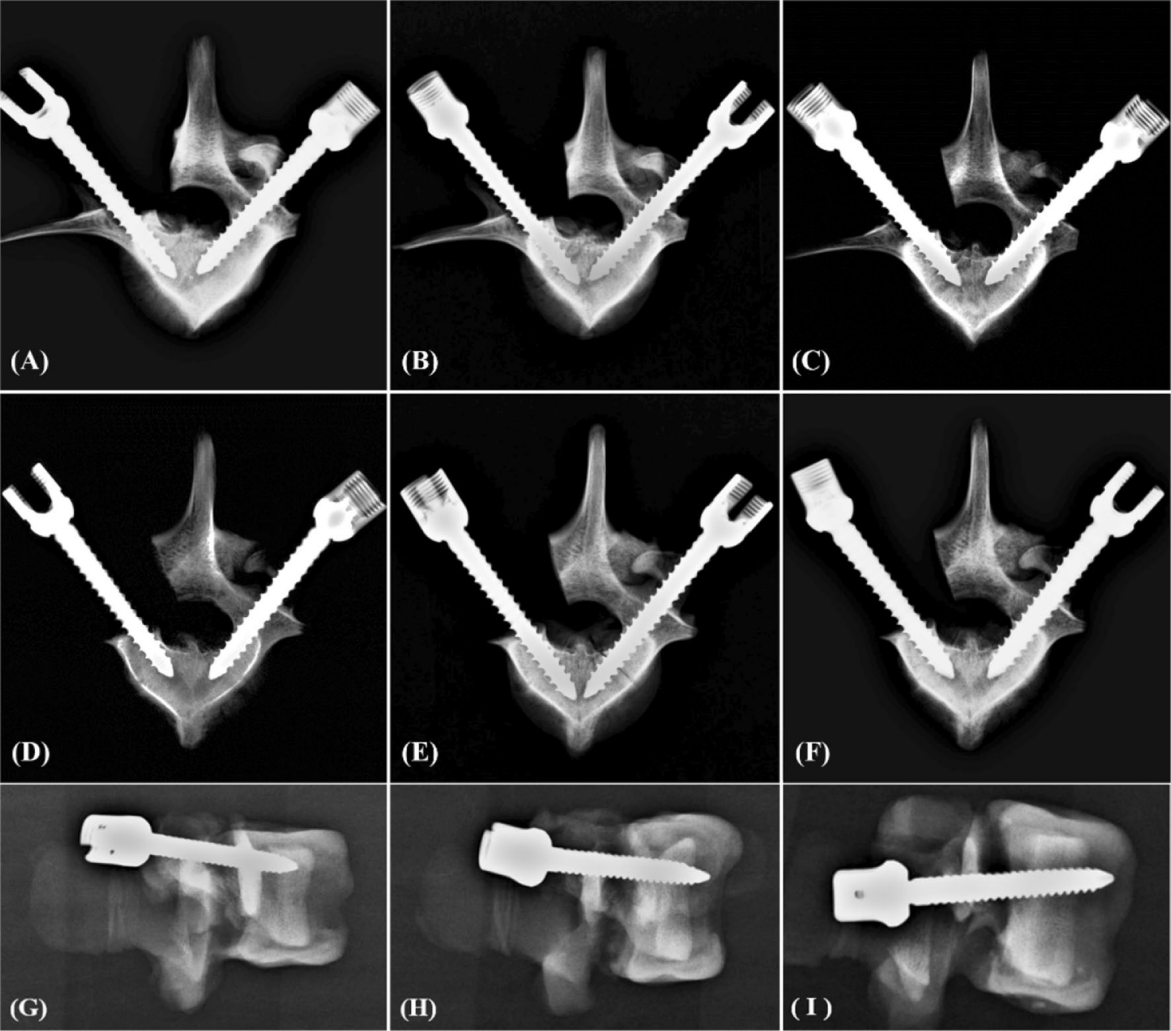
X-ray images showing screw trajectory and insertional depth in the normal-density group. An appropriate screw trajectory and insertional depth were confirmed by the axial view (**A**–**F**) and sagittal view (**G**–**I**). Intact (right-side screws in **A**–**F**), semi-pedicle (left-side screws in **A**–**C**) and non-pedicle (left-side screws in **D**–**F**) modalities for all screws were inserted convergently into the vertebral body, and an additional 5 mm of depth was clearly observed in the 6.0 × 50-mm screw (**B**, **E**, **H**) compared with the other screws (**A**, **C**, **D**, **F**, **G**, **I**). The difference in pedicles between the three groups cannot be distinguished in the sagittal view (**G**–**I**). No fractures or defects in the vertebrae were detected in either view.

**Figure 3:**
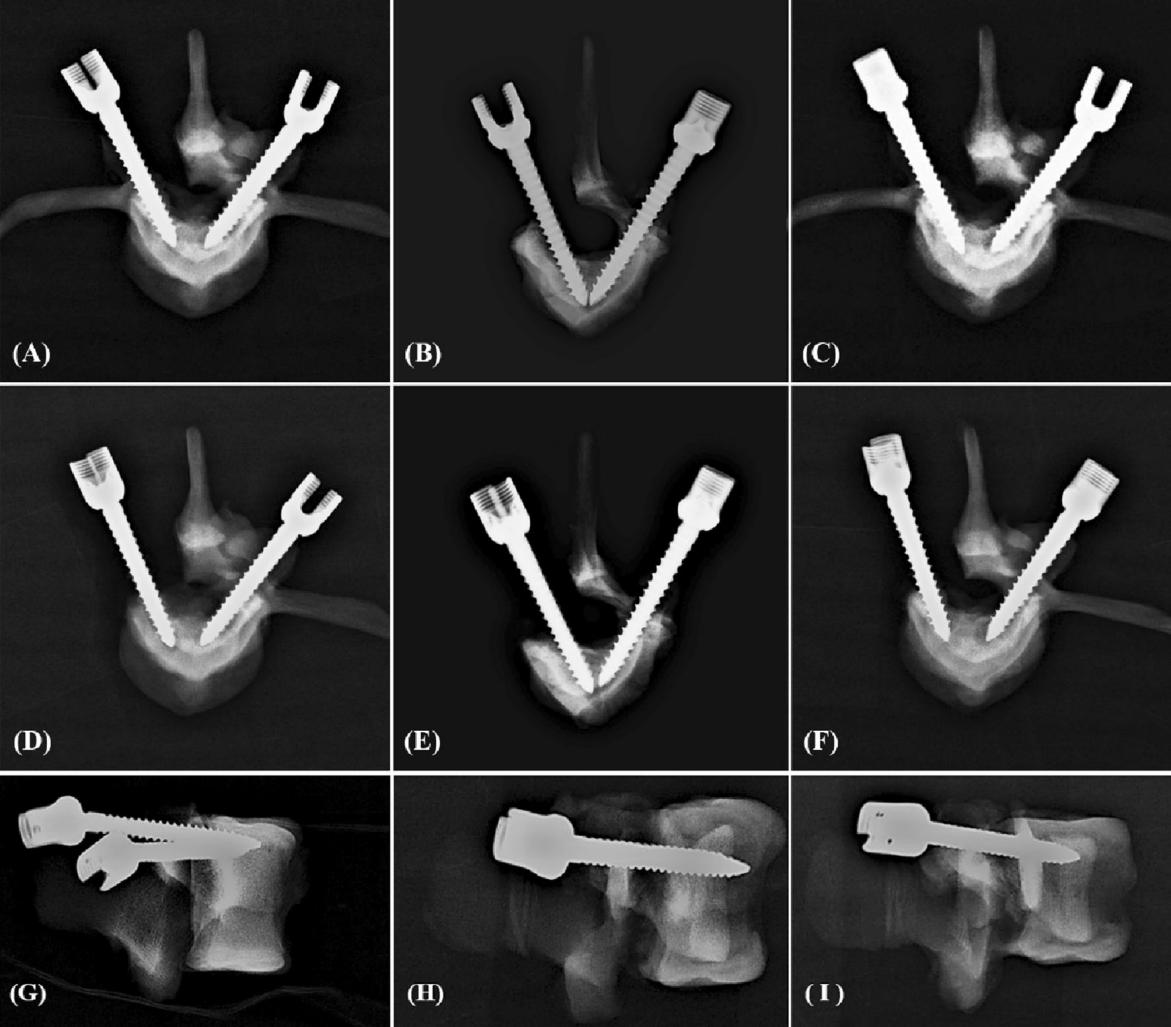
X-ray images showing screw trajectory and insertional depth in the osteoporosis group. An appropriate screw trajectory and insertional depth were confirmed in the axial view (**A**–**F**) and sagittal view (**G**–**I**). Intact (right-side screws in **A**–**F**), semi-pedicle (left-side screws in **A**–**C**) and non-pedicle (left-side screws in **D**–**E**) modalities for all screws were convergently inserted into the vertebral body, and an additional 5 mm of depth was clearly observed for the 6.0 × 50-mm screws compared with the other screws (**A**, **C**, **D**, **F**). The semi-pedicles in the osteoporosis group (Fig. 3**A**–**C**) were not as clear as those in the normal-density group (Fig. 2**A**–**C**) because of the decalcified effect.

An appropriate screw trajectory and insertional depth were confirmed using X-ray imaging prior to pullout testing in the normal-density group (Fig. 2) and osteoporosis group (Fig. 3). Intact (Figs. 2, 3, all right-side screws in axial views), semi-pedicle (Figs. 2, 3A–C, left-side screws) and non-pedicle (Figs. 2, 3D–F, left-side screws) modalities for all screws were convergently inserted into the vertebral body, and an additional 5 mm of depth was clearly observed in the 6.0 × 50-mm group (Figs. 2, 3, B, E, H) compared with the other modalities. The semi-pedicles in the osteoporosis group (Fig. 3A–C) were not as clear as those in the normal-density group (Fig. 2A–C) because of the decalcified effect. No fractures or defects in the vertebrae were detected in either view.

### Pullout strength

**Figure 4:**
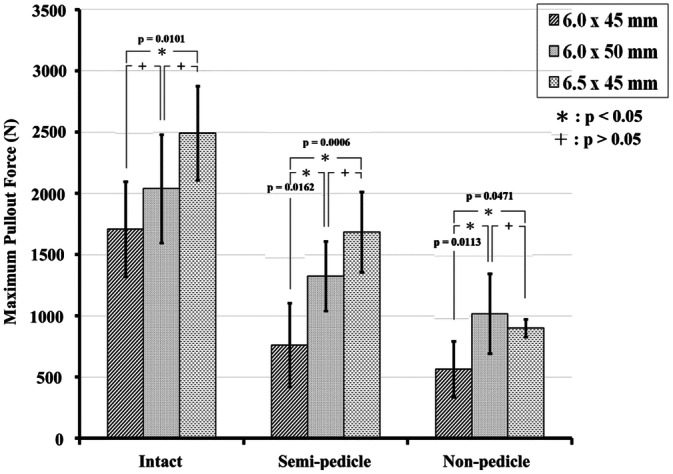
Mean ultimate pullout forces for various screw sizes and pedicle modalities in the normal-density group. Compared with the 6.0 mm × 45 mm screw (primary), the pullout force of larger-diameter screws (6.5 mm × 45 mm) was significantly higher in all pedicle modalities, whereas the pullout force in longer screws (6.0 mm × 50 mm) was significantly higher in the semi- and nonpedicle modalities.

In the normal-density groups, 6.0 mm × 45 mm, 6.0 mm × 50 mm, and 6.5 mm × 45 mm screws had mean pullout strength values of 1,710.1 ± 387.6 N, 2039.1 ± 443.1 N, and 2,493.1 ± 383.2 N, respectively, in the intact pedicle modality; 762.6 ± 341.8 N, 1,325.1 ± 282.6 N, and 1686.1 ± 327.4 N, respectively, in the semi-pedicle modality; and 565.2 ± 226.9 N, 1,019.4 ± 324.8 N, and 901.3 ± 127.6 N, respectively, in the non-pedicle modality (Fig. 4; Supplementary Table S1). Compared with the 6.0 mm × 45 mm-screw (primary), the pullout force of larger-diameter screws (6.5 mm × 45 mm) was significantly higher in all pedicle modalities, whereas the pullout force for longer screws (6.0 mm × 50 mm) was significantly higher in the semi- (p = 0.0162) and non-pedicle modalities (p = 0.0113) (Supplementary Table S2).

**Figure 5:**
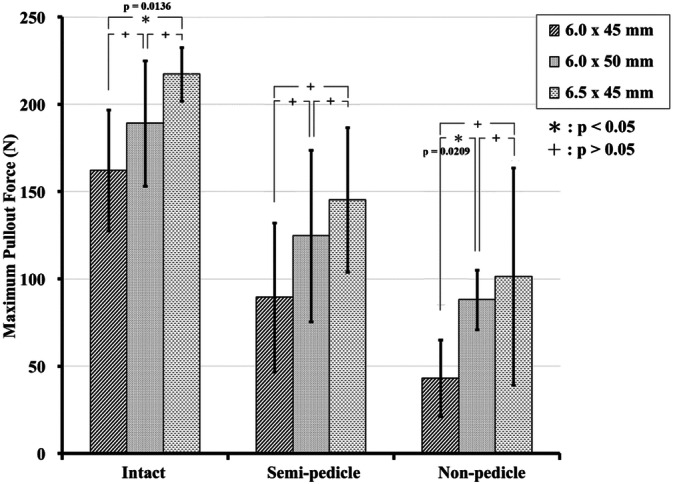
Mean ultimate pullout forces for various screw sizes and pedicle modalities in the osteoporosis group. Compared with the 6.0 mm × 45 mm screw (primary), the pullout force of the larger-diameter screw (6.5 mm × 45 mm) was significantly higher only in the intact pedicle modality; in addition, the pullout force of the longer screw (6.0 mm × 50 mm) was significantly higher only in the non-pedicle modality.

In the osteoporosis groups, the 6.0 mm × 45 mm, 6.0 mm × 50 mm, and 6.5 mm × 45 mm screws had mean pullout strength values of 162.2 ± 34.7 N, 189.1 ± 36.0 N, and 217.3 ± 15.3 N, respectively, in the intact pedicle modality; 89.5 ± 42.6 N, 124.7 ± 49.1 N, and 145.4 ± 41.4 N, respectively, in the semi-pedicle modality; and 43.1 ± 22.0 N, 88.2 ± 17.1 N, and 101.4 ± 62.1 N, respectively, in the non-pedicle modality (Fig. 5; Supplementary Table S3). Compared with the 6.0 mm × 45-mm screw (primary), the pullout force of the larger-diameter screw (6.5 mm × 45 mm) was significantly higher only in the intact pedicle modality (p = 0.0136); in addition, the pullout force of the longer screw (6.0 mm × 50 mm) was significantly higher only in the nonpedicle modality (p = 0.0209) (Supplementary Table S4).

**Figure 6:**
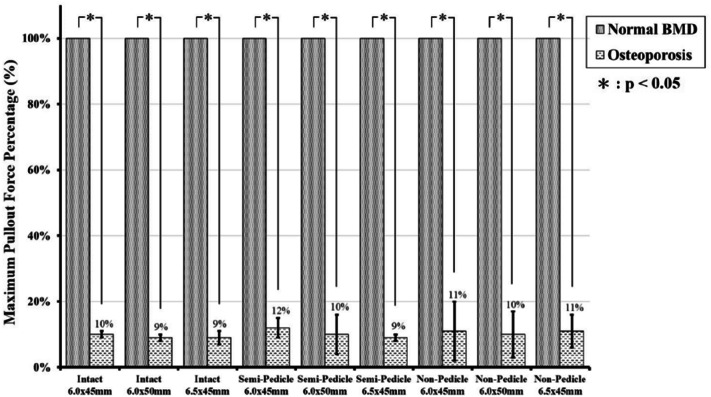
Maximum pullout force in the osteoporosis group, presented as a percentage compared with the normal-density group. Only a 9–12% pullout force was preserved in the osteoporosis groups.

A comparison of the pullout force between the normal-density group and the osteoporosis group for various screw sizes and pedicle modalities is shown in Fig. 6. The maximal pullout force in the osteoporosis group was significantly lower than that in the normal density group, reaching only 9–12% for the same screw size and pedicle modality.

**Figure 7:**
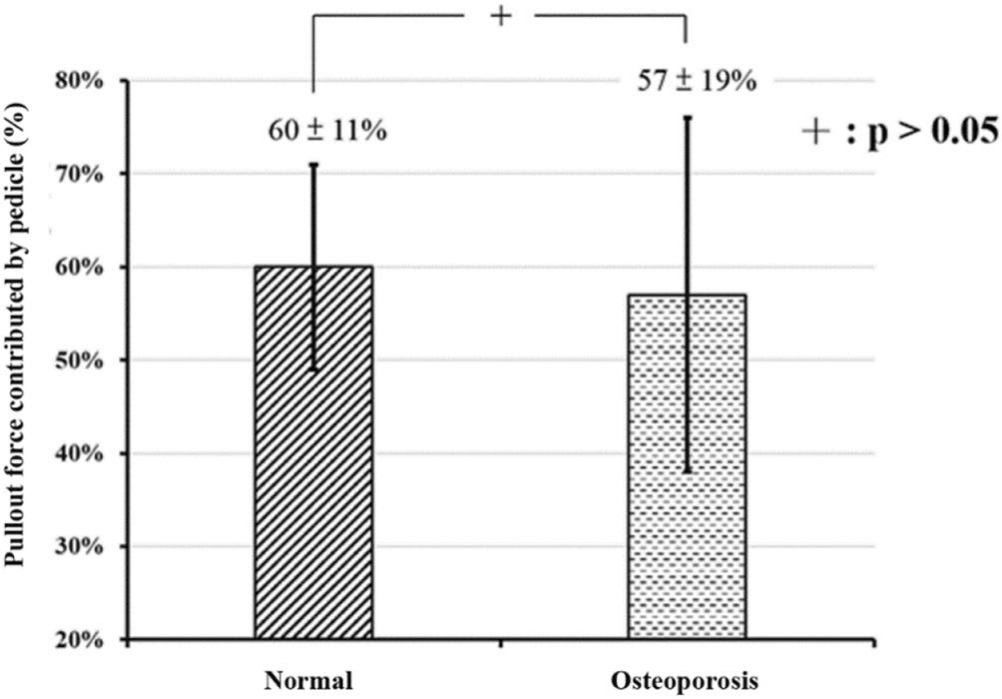
The pullout force percentage contributed by the pedicle in the normal-density and osteoporosis groups. The pullout force contributed by the pedicle was 60 ± 11% and 57 ± 19% in the normal and osteoporosis groups, respectively. No significant difference between groups was found (*p* < 0.05).

The pullout force percentage contributed by the pedicle for the normal and osteoporotic groups is shown in Fig. 7. The pullout force percentage of the pedicle in either the normal or osteoporotic group is calculated as follows:

$$ {\text{Percentage}}\;{\text{ of}}\;{\text{ pullout }}\;{\text{force}} = \frac{{({\text{MPF }}\;{\text{in }}\;{\text{intact}} - {\text{MPF }}\;{\text{in }}\;{\text{nonpedicle}})}}{{{\text{MPF}}\;{\text{ in }}\;{\text{intact}}}} $$

where “MPF” denotes the maximum pullout force.

The pullout force percentage of the pedicle was 60 ± 11% in the normal-density group and 57 ± 19% in the osteoporosis group, but the difference was not significant (Fig. 7). Only 40% and 43% pullout strength were retained after pedicles were broken in the normal-density group and osteoporosis group, respectively. In our previous study, in which a standard sawbone was used to investigate the screw pullout strength contributed by the pedicle^4^, the results revealed that pullout strengths of only 41–45% in the semipedicle group and 29–39% in the nonpedicle group were retained for all three screw sizes. These results indicate that the pullout force percentage contributed by the pedicle reaches 59–71%. The pullout force contributed by the pedicle seems to be higher in standard sawbone than in porcine vertebrae.

## Discussion

The primary goal of this study was to determine any difference in pullout force contributed by the pedicle between the normal bone density and osteoporosis groups. We used L4 porcine vertebrae with different bone densities instead of standard L4 sawbones to simulate true clinical applications.

The availability of fresh frozen human cadavers is very limited, and the porcine spine can be a good alternative model because of the similar geometric characteristics of the vertebral body, spinal canal, and pedicle shape^11,12^. L4 porcine vertebrae shared a more similar pedicle width with humans compared with goats, sheep and dogs^13^; meanwhile, a study demonstrated similar biomechanical results between formalin-fixated specimens and fresh frozen cadavers^14^.

**Figure 8:**
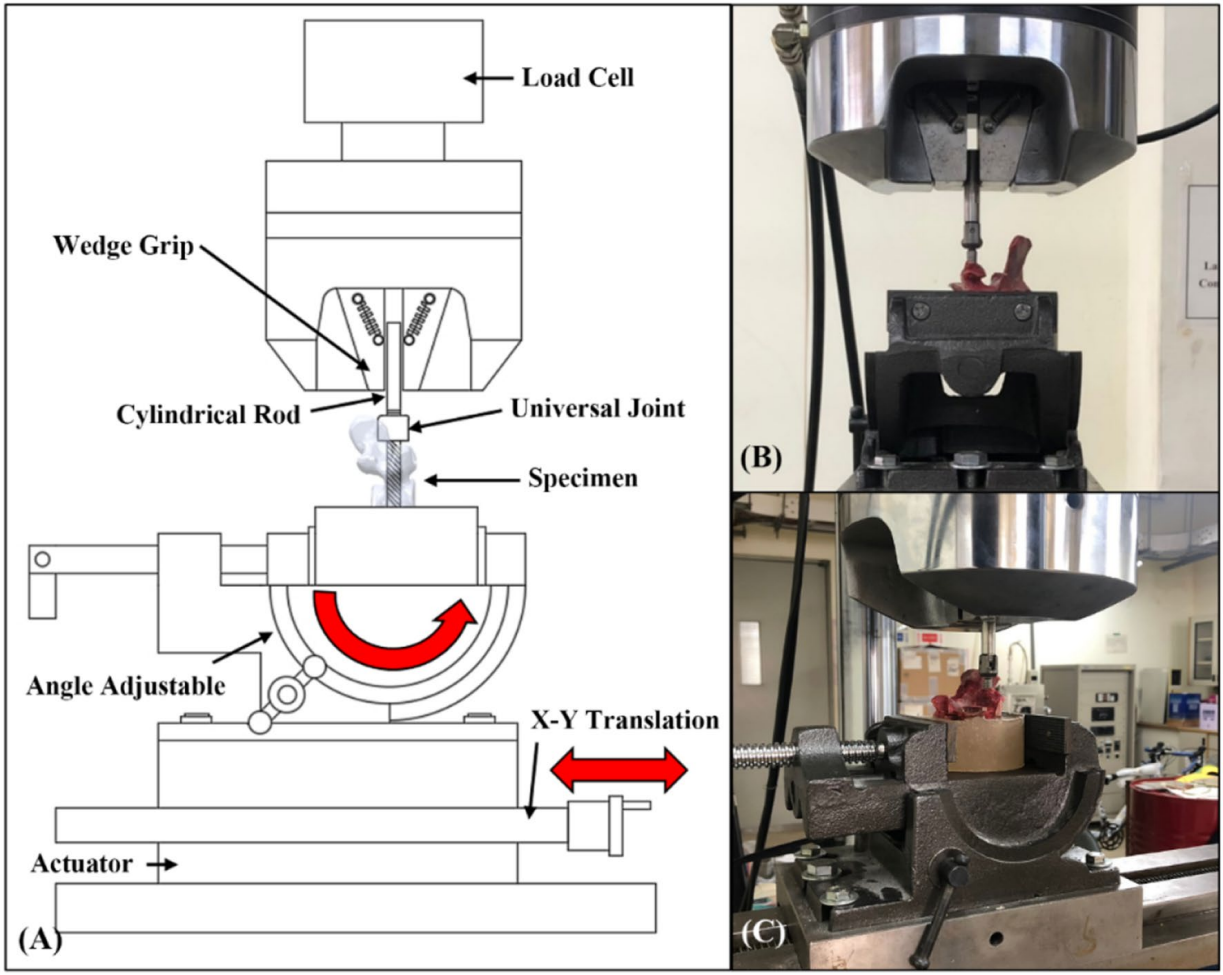
Schematic drawing and photograph showing the screw pullout test in a vertebral specimen. The polyaxial screw head was fixed to a 10-mm diameter cylindrical rod with an outer thread that matched the inner thread of the screw head via the universal head (**A**). The cylindrical rod was then clamped to the upper wedge grip of the MTS testing machine (**A**). The potted specimen was secured on a lower custom-made grip capable of x–y plane (**A**, red double arrowheads) translation and rotation (**A**, red curved arrow) to achieve coaxial alignment of the pedicle screw with the pullout ram. Front view (**B**) and lateral view (**C**) showed that the screws and the direction of the pullout force were along the same axis.

**Figure 9:**
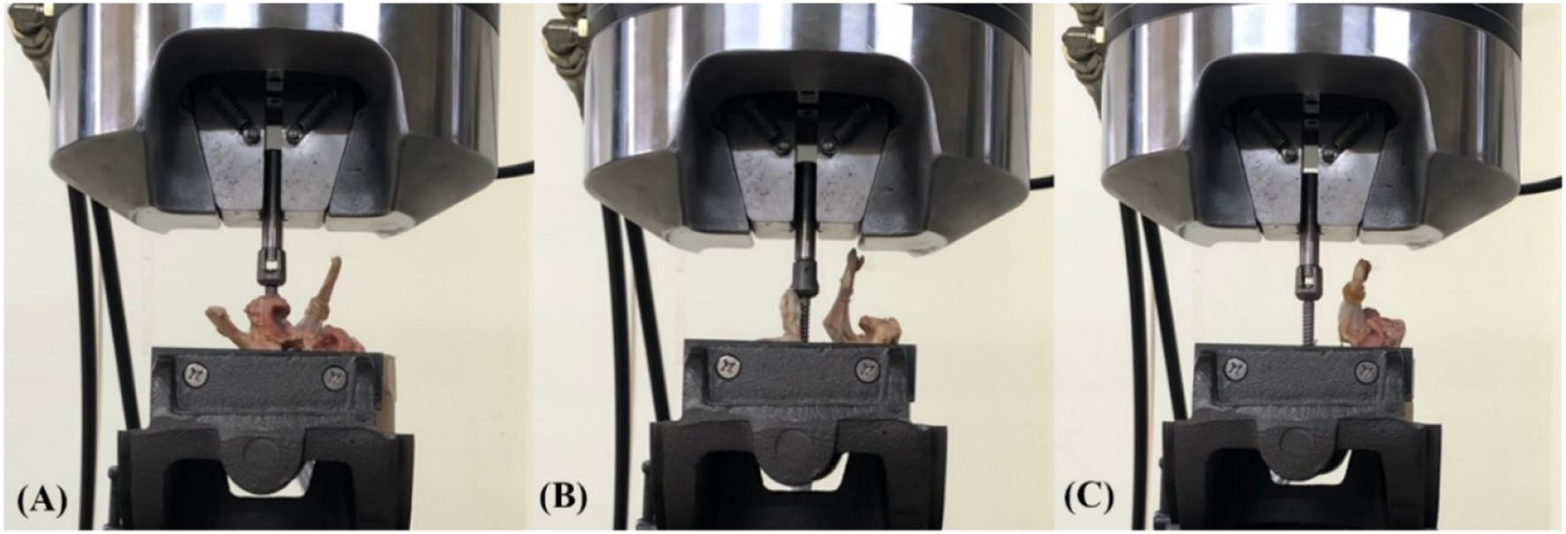
Photograph showing the screw pullout test of vertebral specimens in an intact pedicle (**A**), a semipedicle (**B**), and a nonpedicle (**C**). All specimens were potted in metal boxes using specific epoxy resins without any screw-cement contact. The pedicle screws and the pullout force were directed along the same axis without deviation.

Interpretation of the pedicle screw fixation in an osteoporosis spine is clinically practical^15–17^. In our study, decalcified porcine vertebrae reaching a BMD < 0.57 g/cm^2^ of the normal bone density (Fig. 1) were allocated to the osteoporosis group^8^. In this study, we used a lower custom-made grip capable of x–y plane translation and rotation (Figs. 8, 9) to achieve coaxial alignment of the pedicle screw with the pullout ram. We ensured that the screws and the direction of the pullout force were along the same axis to prevent an 8% error by mounting the screws onto a material testing machine at different angles^9^.

**Figure 10:**
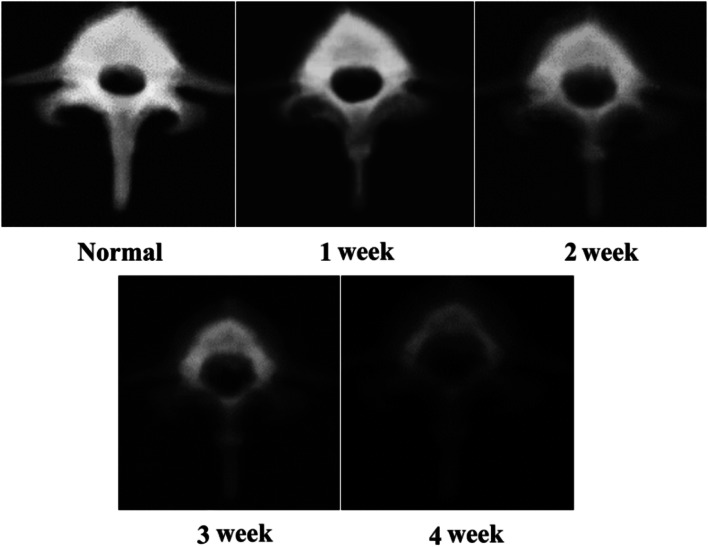
The vertebrae underwent X-ray imaging every week during the decalcification process. Progressive radiolucency over the vertebral body and pedicle was observed during the decalcification process from the first week to the 4th week.

In the normal-density group, the pullout forces of the larger-diameter screws (6.5 mm × 45 mm) and longer screws (6.0 mm × 50 mm) were significantly higher than those of the 6.0 mm × 45 mm screws in the semi- and non-pedicle modalities, which means that switching to larger-diameter screws or longer screws could be a salvage method when the pedicle is broken during surgery in individuals with normal bone density. However, in the osteoporosis group, the significantly higher pullout force in the non-pedicle modality belonged only to the longer screw, which implies that the use of only a longer screw can be considered a means of salvaging. Reasons for the difference in salvage conditions between the normal bone density and osteoporosis groups are the following: (1) a result of the bone density decreasing from the surroundings to the center of the vertebral body during the decalcification process, which implies that a longer screw rather than a larger-diameter screw would be suitable for the inner undecalcified bone (Fig. 10); and (2) pullout strength was created by friction force between the implant and engaged bone^18–20^, which was higher for larger diameter screws in the normal-density group, but decreased cancellous bone in the osteoporosis group reduce the engaged bone volume and, thus, alleviated the effect of the larger diameter screw.

In our study, the pullout force decreased to 9–12% of that of the osteoporotic group (Fig. 6), which is not comparable with other studies. Schulze et al.^21^ reported that the 503 N (279/731) pullout force in the osteoporotic human spine, which is significantly larger than that found in our study in the osteoporosis group, could be explained using different pedicle contributions and different pedicle anatomical sizes between humans and swine^22^. Lee et al.^8^ stated that approximately 16.02% of the mean compression ultimate strain in similar osteoporotic vertebrae is conducted in a biomechanical axial compression system instead of a pullout test. The similar decrease in BMD did not imply the corresponding reduction in strength due to the use of different anatomical sites for measurement and testing methods. Lai et al.^23^ reported that larger diameter screws (5.0 mm × 35 mm) achieved a higher pullout strength than that of smaller (4.35 mm × 35 mm) screws in an osteoporotic model. The osteoporotic density was not uniform, and tests were performed in human thoracic vertebrae, yielding different results than those of this study.

In our normal-density porcine vertebrae, the average pullout strength after the complete percentage of the pedicle contributed to the pullout force reached 40% (Fig. 7), which is slightly higher than the pullout force (29–39%) in the nonpedicles of sawbones^4^ because of the different pedicle sizes between humans and swine^11,22^. A higher pullout force contributed by the pedicle in sawbones is predictable because the pedicle width and height are significantly larger in humans than in pigs^11^.

To the best of the authors’ knowledge, no biomechanical reports have addressed the pullout strength of the pedicle in a normal BMD and osteoporotic porcine vertebral model or the subsequent salvage strategies to prevent screw loosening. The percentage of pullout force contributed by the pedicle reached 60% in the normal-density group and 57% in the osteoporosis group, without a significant difference, which indicates the important role of the pedicle in either normal bone density or osteoporotic vertebrae through the whole screw pullout performance. Due to the preserved force of only 9–12% after decalcification, longer screws with supplementary strengthening materials^24–29^ or methods^30–32^ should be applied clinically, especially when pedicles are broken in osteoporotic spines.

The present study had some limitations. First, inherent limitations, such as different geometries or density distributions of porcine vertebra compared with those of humans may limit clinical practice^12,33^. Second, the lack of blindness of practitioners, data collectors and analysts could result in potential selection bias. Third, limited number of specimens might increase variability of data and reduce the statistical reliability. Finally, data from standard experimental semi-pedicles and non-pedicles may not completely reflect clinical biomechanical data from irregular-shaped broken pedicles.

## Conclusions

The pedicle plays an important role in both the normal bone density group and the osteoporosis group on the basis of the whole screw pullout performance. In the normal-density group, both longer and larger-diameter screws could be used for salvaging; however, in the osteoporosis group, only the use of a longer screw would be a salvage method.

## Materials and methods

**Figure 11:**
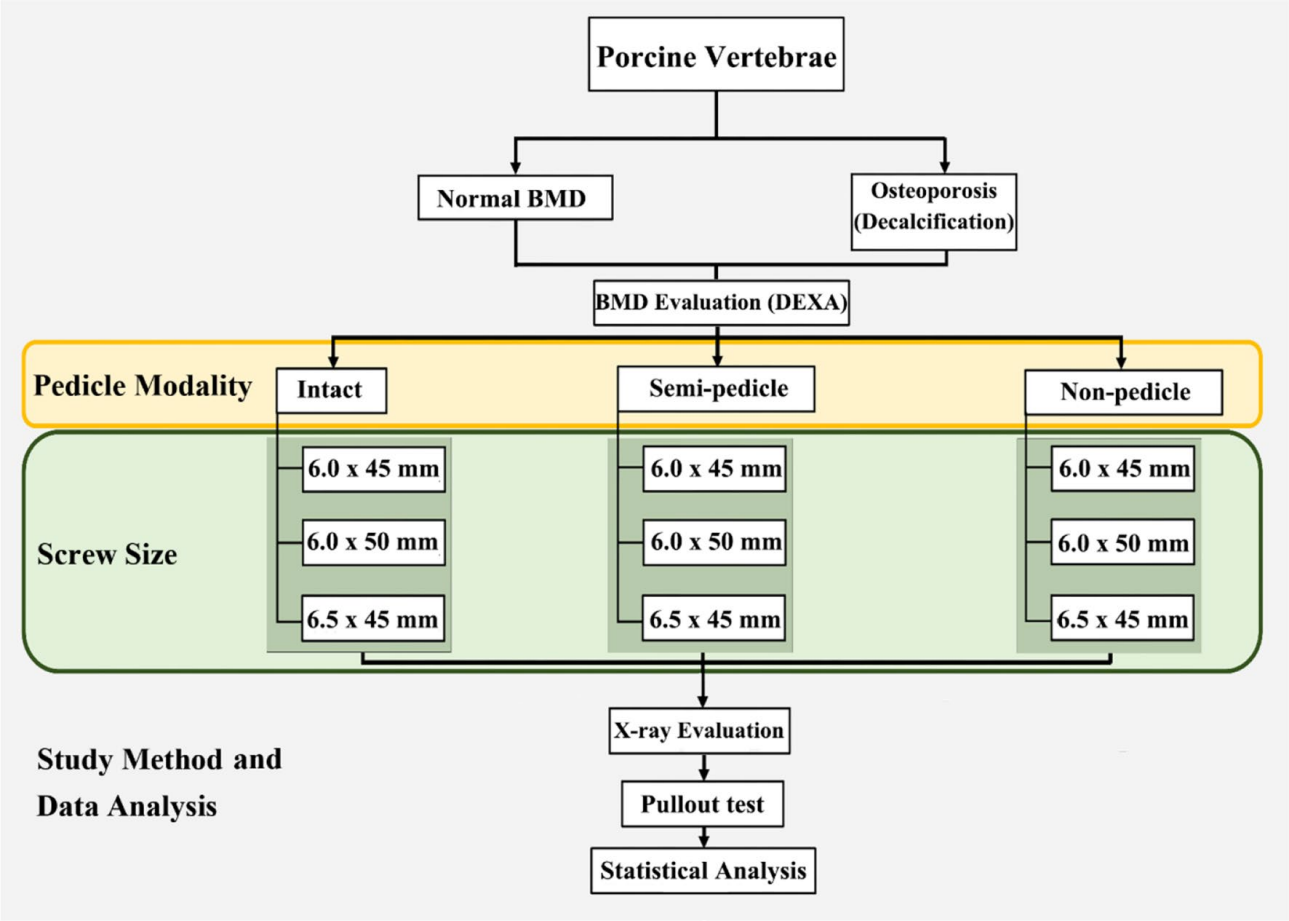
A flowchart of the study design.

This study was approved by the review board of Chang Gung Memorial Hospital (CRRPG3H0082). All experiments conformed to the regulations for the care and use of animals. The usage of the porcine spines was in accordance with the guidelines of replacement, reduction and refinement. A flowchart of the study design is presented in Fig. 11.

### Specimen preparation and decalcified method

This study was performed using sixty L4 fresh-frozen lumbar vertebrae harvested from mature pigs (weight 100–120 kg; age 12–18 months). All animals were healthy before harvesting and never exposed to any drugs or procedures that could affect the bone density. All specimens were separated into individual vertebrae after the surrounding musculature, ligaments, and periosteum were stripped off. Thirty of these 60 vertebrae were designed as the normal-density group and embedded in 10% formalin solution (SIGMA-HT501640, Sigma Chemical Co.) for 24 h before the biomechanical and imaging studies. The other 30 specimens were designed as the osteoporosis group and stored in a glass tank at room temperature for four weeks of decalcification. The glass tanks were filled with 500 ml, 0.5 Mof EDTA decalcification solution (pH 7.4, Sigma), with renewal of the EDTA (SIGMA-E5134, Sigma Chemical Co.) every week after embedding in a 10% formalin solution for 24 h.

### X-ray image and bone mineral density during decalcification

X-ray images (RS Safire, Shimadzu Co.) were obtained every week during the decalcification process (Fig. 10). The post-processing BMD (bone mineral density) was measured and recorded every week by a dual-energy X-ray absorptiometer (QDR-4500, Hologic) by the same technician during the entire decalcification process.

### Specimen grouping and screw insertion

**Figure 12:**
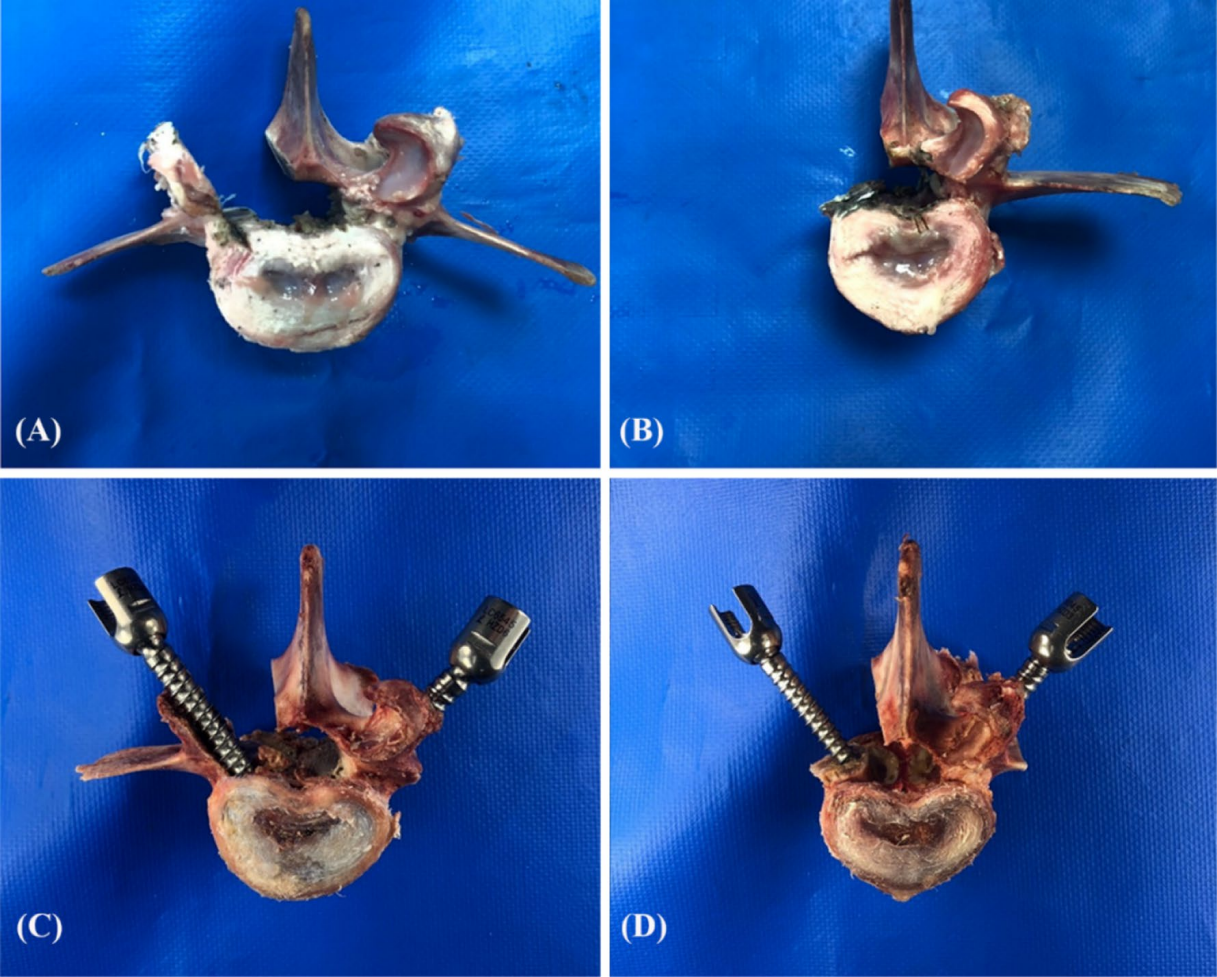
Photograph showing various pedicle modalities. Porcine vertebrae were designed to represent semi-pedicles (**A**, left), non-pedicles (**B**, left), and intact pedicles (**A**, **B**, right). Screw insertions in the intact pedicle (**C**, **D** right), semi-pedicle (**C**, left) and non-pedicle (**D**, left) are shown in the lower row.

The specimens were divided into two groups: normal-density and osteoporosis; each group was designed to represent an intact pedicle, a semipedicle, or a nonpedicle modality^4^. A pilot hole was drilled using a 2.5 mm “twist” metric drill bit attached to a Dremel 4000 rotary tool that was mounted on a Dremel WorkStation Model 220-01. This trajectory was selected based on previously reported morphometric characteristic data^33^. The pilot track was followed with a standard straight pedicle probe to a depth of 45 mm. Three sizes of polyaxial screws (diameter × length of 6.0 mm × 45 mm, 6.0 mm × 50 mm, and 6.5 mm × 45 mm) (SmartLoc spinal polyaxial pedicle screws, A-spine Asia Co. Ltd., Taipei, Taiwan) were chosen and randomly implanted into each pedicle of the vertebrae by an experienced surgeon. These chosen screw sizes were based on the most commonly used clinically. Then, the specimens were randomly distributed into the intact pedicle modality (n = 30), the semipedicle modality (n = 15), or the nonpedicle modality (n = 15). These experiments were conducted over five trials for each screw size (Fig. 12). Before the pullout test, the specimens and screws were closely examined for signs of fracture and damage and any findings were carefully recorded.

### Pullout test

Each of the 60 specimens was potted in metal boxes using specific epoxy resins (Buehler, Lake Bluff, IL, USA). Judicious potting was performed to ensure that the cement did not come into contact with any portion of the pedicle screw. The prepared specimens were mounted onto a material testing machine (Bionix 858; MTS Systems Corp., MN, USA) to conduct axial pullout tests with the screws (Fig. 8). The polyaxial screw head was fixed to a cylindrical rod with an outer thread that matched the inner thread of the screw head via the universal head (Fig. 8A). The cylindrical rod was then clamped to the upper wedge grip of the MTS testing machine. The potted specimen was secured on a lower custom-made grip capable of x–y plane (Fig. 8A, red double arrowheads) translation and rotation (Fig. 8A, red curved arrow) to achieve coaxial alignment of the pedicle screw with the pullout arm. We ensured that the pedicle screws and the pullout force were directed along the same axis by clamping the potted specimen in a way that enabled free translation in the x–y plane and free rotation about the axis, and the direction of the pullout force could be adjusted through the polyaxial design of the screw head. Different views (Fig. 8B, C) showed that the screws and the direction of the pullout force were along the same axis. Images confirmed that no deviation of the axis occurred in the three different pedicle modalities (Fig. 9). The screws were then loaded in displacement control mode at a constant displacement rate of 5 mm/min for a total displacement of 10 mm, which is in accordance with published literature on axial pullout testing^34,35^. Data collection was set at 1 sample/0.05 mm (1.67 Hz). Failure was defined as the maximum load or the load peak prior to a decrease in load associated with increasing displacement^36^. After the pullout test was completed, the specimen and the screws were closely examined for signs of fracture and damage and any findings were carefully recorded.

### Statistics

Statistical software (SPSS for Windows version 12.0, SPSS Inc., Chicago, IL, USA) is used to analyze the pullout force of all specimens. All of the measurements were collected from 60 vertebrae and expressed as the mean ± standard deviation. Normal distribution of the sample was examined using Shapiro–Wilk test; whereas Levene's test was used to test the Homogeneity of Variances. ANOVA test with post hoc LSD analysis was performed to evaluate the differences between groups if the data is normally distributed and homogeneity. Differences were considered significant at *p* < 0.05.

## Supplementary information

Supplementary information.
