# Supplementary material for: Use of longer sized screws is a salvage method for broken pedicles in osteoporotic vertebrae
Source: Sci Rep. 2020 Jun 26;10:10441. doi: 10.1038/s41598-020-67489-2 (PMC7320151; doi:10.1038/s41598-020-67489-2)
Supplement: Supplementary file 1 — Supplementary information. [file 41598_2020_67489_MOESM1_ESM.docx]

**Supplementary Information**

**Use of longer sized screws is a salvage method for broken pedicles in osteoporotic vertebrae**

Ming-Kai Hsieh^1,2,3^, mk660628@gmail.com

Mu-Yi Liu^4^, zero_790723@hotmail.com

Jin-Kai Chen^5^, sh25605382@gmail.com

Tsung-Ting Tsai^2,3^, tsai1129@gmail.com

Po-Liang Lai^2,3^, polianglai@gmail.com

Chi-Chien Niu^2,3^, niuchien@adm.cgmh.org.tw

Ching-Lung Tai^2,3,5*^, taicl@mail.cgu.edu.tw

*****Correspondence: **taicl@mail.cgu.edu.tw**

^1^ Institute of Biotechnology, National Taiwan University, Taipei, Taiwan

^2^ Bone and Joint Research Center, Chang Gung Memorial Hospital, Taoyuan, Taiwan

^3^ Department of Orthopaedic Surgery, Spine Section, Chang Gung Memorial Hospital and College of Medicine, Chang Gung University, Taoyuan, Taiwan

^4^ Ph.D. Program in Biomedical Engineering, Collage of Engineering, Chang Gung University, 33302, Taoyuan, Taiwan

^5^ Graduate Institute of Biomedical Engineering, Chang Gung University, Taoyuan, Taiwan

**Table S1: Ultimate Pullout Forces for various screw sizes and pedicle modalities in the Normal-density group.**

|  | **Ultimate Pullout Force (N) in the Normal-density group** | | | | | | | | |
| --- | --- | --- | --- | --- | --- | --- | --- | --- | --- |
| **Specimen No.** | **Intact**  **6.0x45 mm** | **Semi-Pedicle**  **6.0x45 mm** | **Non-Pedicle**  **6.0x45 mm** | **Intact**  **6.0x50 mm** | **Semi-Pedicle**  **6.0x50 mm** | **Non-Pedicle**  **6.0x50 mm** | **Intact**  **6.5x45 mm** | **Semi-Pedicle**  **6.5x45 mm** | **Non-Pedicle**  **6.5x45 mm** |
| **1** | 2070.2 | 1155.0 | 840.2 | 2379.1 | 1578.8 | 1266.4 | 2896.0 | 2138.8 | 1044.6 |
| **2** | 1883.3 | 989.5 | 653.7 | 2202.3 | 1560.2 | 1225.3 | 2607.4 | 1714.7 | 973.0 |
| **3** | 1439.2 | 781.6 | 347.8 | 1791.3 | 1097.8 | 892.9 | 2221.0 | 1311.9 | 799.6 |
| **4** | 1170.5 | 273.3 | 312.6 | 1387.4 | 955.6 | 503.3 | 1978.1 | 1436.0 | 738.2 |
| **5** | 1987.3 | 613.7 | 671.7 | 2435.2 | 1433.1 | 1209.4 | 2762.9 | 1829.3 | 951.3 |
| **Average** | 1710.1 | 762.6 | 565.2 | 2039.1 | 1325.1 | 1019.4 | 2493.1 | 1686.1 | 901.3 |
| **SD** | 387.6 | 341.8 | 226.9 | 443.1 | 282.6 | 324.8 | 383.2 | 327.4 | 127.6 |

**Table S2: Exact p-values between groups for various screw sizes and pedicle modalities in the Normal-density group.**

| **Exact p-values between groups in the Normal-density group** | | | |
| --- | --- | --- | --- |
| **Intact** |  | **6.0 mm×45 mm** | **6.0 mm×50 mm** |
|  | **6.0 mm×50 mm** | 0.223848606 | - |
|  | **6.5 mm×45 mm** | 0.010033859 | 0.102087986 |
|  |  |  |  |
| **Semi-Pedicle** |  | **6.0 mm×45 mm** | **6.0 mm×50 mm** |
|  | **6.0 mm×50 mm** | 0.016216142 | - |
|  | **6.5 mm×45 mm** | 0.000624034 | 0.098116731 |
|  |  |  |  |
| **Non-Pedicle** |  | **6.0 mm×45 mm** | **6.0 mm×50 mm** |
|  | **6.0 mm×50 mm** | 0.011296502 | - |
|  | **6.5 mm×45 mm** | 0.047140468 | 0.452039553 |

**Table S3: Ultimate Pullout Forces for various screw sizes and pedicle modalities in the Osteoporotic group.**

|  | **Ultimate Pullout Force (N) in the Osteoporotic group** | | | | | | | | |
| --- | --- | --- | --- | --- | --- | --- | --- | --- | --- |
| **Specimen No.** | **Intact**  **6.0x45 mm** | **Semi-Pedicle**  **6.0x45 mm** | **Non-Pedicle**  **6.0x45 mm** | **Intact**  **6.0x50 mm** | **Semi-Pedicle**  **6.0x50 mm** | **Non-Pedicle**  **6.0x50 mm** | **Intact**  **6.5x45 mm** | **Semi-Pedicle**  **6.5x45 mm** | **Non-Pedicle**  **6.5x45 mm** |
| **1** | 172.4 | 105.5 | 68.3 | 200.7 | 154.4 | 95.1 | 222.3 | 162.4 | 135.2 |
| **2** | 189.5 | 149.1 | 62.3 | 232.3 | 167.8 | 113.0 | 228.2 | 205.8 | 191.6 |
| **3** | 181.3 | 95.4 | 41.3 | 193.3 | 157.5 | 83.0 | 210.2 | 136.4 | 84.7 |
| **4** | 165.5 | 56.7 | 25.0 | 186.4 | 84.0 | 82.6 | 231.6 | 126.8 | 55.4 |
| **5** | 102.3 | 41.0 | 18.5 | 133.0 | 60.1 | 67.1 | 194.2 | 95.6 | 40.1 |
| **Average** | 162.2 | 89.5 | 43.1 | 189.1 | 124.7 | 88.2 | 217.3 | 145.4 | 101.4 |
| **SD** | 34.7 | 42.6 | 22.0 | 36.0 | 49.1 | 17.1 | 15.3 | 41.4 | 62.1 |

**Table S4: Exact p-values between groups for various screw sizes and pedicle modalities in the Osteoporotic group.**

| **Exact p-values between groups in the Osteoporotic group** | | | |
| --- | --- | --- | --- |
| **Intact** |  | **6.0 mm×45 mm** | **6.0 mm×50 mm** |
|  | **6.0 mm×50 mm** | 0.183420721 | - |
|  | **6.5 mm×45 mm** | 0.013634992 | 0.1657213 |
|  |  |  |  |
| **Semi-Pedicle** |  | **6.0 mm×45 mm** | **6.0 mm×50 mm** |
|  | **6.0 mm×50 mm** | Nonsignificant among three groups (p = 0.176146) | |
|  | **6.5 mm×45 mm** |  |  |
|  |  |  |  |
| **Non-Pedicle** |  | **6.5 mm×45 mm** | **6.0 mm×50 mm** |
|  | **6.0 mm×50 mm** | 0.020879349 | - |
|  | **6.5 mm×45 mm** | 0.250462957 | 0.951713507 |
